# Xanthohumol prevents dextran sulfate sodium-induced colitis via inhibition of IKKβ/NF-κB signaling in mice

**DOI:** 10.18632/oncotarget.23183

**Published:** 2017-12-12

**Authors:** Jae-Min Cho, Sun-Mi Yun, Young-Ho Choi, Jinyuk Heo, Nam-Jung Kim, Seok-Ho Kim, Eun-Hee Kim

**Affiliations:** ^1^ College of Pharmacy and Institute of Pharmaceutical Sciences, CHA University, Seongnam 13488, Korea; ^2^ College of Pharmacy, Kyung Hee University, Seoul 02447, Korea

**Keywords:** xanthohumol, DSS, colitis, NF-κB, IKKβ

## Abstract

Xanthohumol (XN), a prenylated chalcone isolated from the hop plant, has been reported to exhibit multiple biological functions including anti-inflammation. However, the pharmacological function of XN on colitis remains unknown. In this study, we investigated the anti-inflammatory effect of synthesized XN and molecular mechanism on dextran sulfate sodium (DSS)-induced experimental colitis. XN attenuated the colitis symptoms along with the prevention of colonic lesions after DSS challenge. XN inhibited the production of pro-inflammatory cytokines, oxidative stress and cyclooxygenase-2 expression in DSS-treated mice. Moreover, XN inhibited the phosphorylation of IκBα, the nuclear translocation of NF-κB subunits and the transcriptional activity of NF-κB *in vivo* and *in vitro*. In contrast to XN, isoXN showed much less effects on the kinase activity of IKKβ and IκBα phosphorylation induced by XN in this study, suggesting that an electrophilic carbon center present in XN is critical for the anti-inflammation in colitis, especially inhibition of IKKβ/NF-κB signaling pathway. Consistently, our docking analysis revealed that XN could bind to the active site, presumably at the Cys99 of IKKβ. Taken together, these findings demonstrate a new function of XN to inhibit IKKβ/NF-κB signaling, suggesting XN could be the potential therapeutic agent for the prevention of colitis.

## INTRODUCTION

Ulcerative colitis (UC) is one of the two major forms of inflammatory bowel disease (IBD), a chronic relapsing inflammatory disorder in the gastrointestinal tract [1, 2]. The pathogenesis of UC has not been fully understood but may be related to a combination of genetic susceptibility, immunological abnormality and environmental triggers such as imbalanced diet and pathogenic bacteria [3, 4]. UC is widely known as one of the most important risk factors developing colorectal cancer (CRC) [5]. Recently, several studies reported that UC-associated CRC patients exhibited poorer survival rate, higher histologic grade, and had a greater tumor multiplicity than sporadic CRC patients [5–7]. Therefore, developing an appropriate strategy for the treatment or prevention of UC has been of great interest.

Nuclear factor-κB (NF-κB) is regarded as a crucial regulator of the immune response, which can initiate and amplify inflammation [8, 9]. NF-κB is usually sequestered in the cytoplasm by a family of inhibitory proteins known as inhibitors of NF-κB (IκBs). Upon stimulation, IκBs are phosphorylated by the IκB kinases (IKK) complex, resulting in its degradation [10]. Subsequently, the translocation of NF-κB to the nucleus has occurred, which induces the expression of pro-inflammatory target genes. Therefore, the key mediator for the activation of NF-κB signaling pathway is IKK complex. IKK complex consists of two catalytic kinase subunits (IKKα and IKKβ) and a structural component (NEMO/IKKγ) [11]. The structures of IKKα and IKKβ are very homologous but functionally distinct. IKKβ has been proposed to mediate pro-inflammatory stimuli-induced canonical NF-κB activation whereas IKKα has been thought to be dispensable for canonical NF-κB pathway [12]. NF-κB has known to be the major pathogenic regulator in IBD, such as Crohn’s disease (CD) and UC patients as well as experimental colitis models [13, 14]. The activation of NF-κB has been reported in IBD patients, which promotes the expression of various pro-inflammatory genes, especially strongly aggravates the course of mucosal inflammation [15]. However, the precise role and the molecular mechanism of IKKβ in IBD have not been fully investigated.

Emerging evidence reveals that the use of natural compounds is important for the prevention of diseases, particularly in inflammatory disorders and cancers [16]. Xanthohumol (XN), a prenylated chalcone isolated from the hop plant [17] has multiple biological functions including anti-cancer [18–25], anti-invasion [26], anti-angiogenesis [27] and anti-inflammation [25, 27, 28]. Nevertheless, the biological function of XN on gastrointestinal diseases such as colitis has not yet been examined. In the present study, we demonstrated the anti-inflammatory effect of XN on dextran sulfate sodium (DSS)-induced experimental colitis. Further mechanistic investigations showed that XN ameliorated DSS-induced colitis through inhibition of NF-κB signaling by interacting with an IKKβ.

## RESULTS

### XN inhibited the kinase activity and downstream signaling of IKKβ

**Scheme 1:**
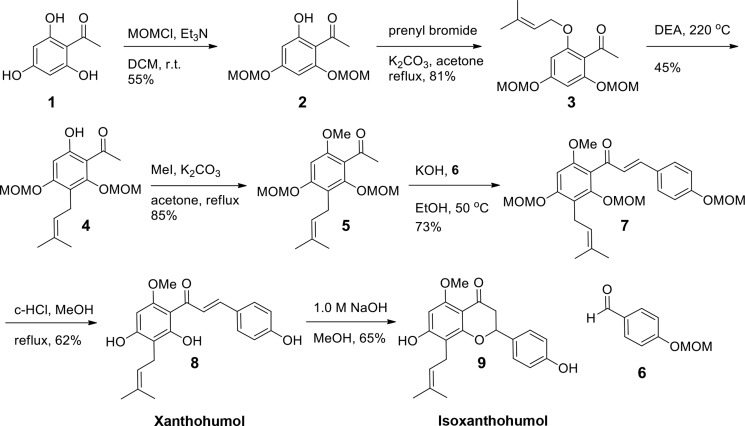
Synthesis of XN and isoXN

To study the function of XN on DSS-induced colitis *in vivo*, we first synthesized enough amount of XN (Scheme 1). Synthesis of XN commenced with commercially available 2, 4, 6-trihydroxy acetophenone based on published procedures [28]. Two hydroxyl groups were protected with methoxymethyl (MOM) ether and remaining hydroxyl group was prenylated by K_2_CO_3_, prenyl bromide in refluxing acetone. Claisen rearrangement of **3** afforded **4** in good yield. In the previous report, *N,N*-diethylaniline was removed by acidic work up procedure, however MOM ether group was very unstable in acidic condition and severe degradation of product was observed [28]. We removed *N*,*N*-diethylaniline by short path distillation in high vacuum and the yield was optimized. The key intermediate **4** was methylated and aldol condensation of **5** with **6** afforded **7** in 73% yield. Deprotection was conducted with catalytic amount of concentrated hydrochloric acid in refluxing methanol in 62% yield. During work up procedure, ethyl acetate and water was added without evaporation of methanol, and two phases were separated because severe cyclization of product was observed during removal of methanol. Isoxanthohumol (isoXN) was also prepared based on known procedure [29]. Treatment of **8** with NaOH solution afforded isoXN, a cyclized product of XN. The ^1^H NMR of **8** and **9** matched with previous reports [28, 29].

**Figure 1:**
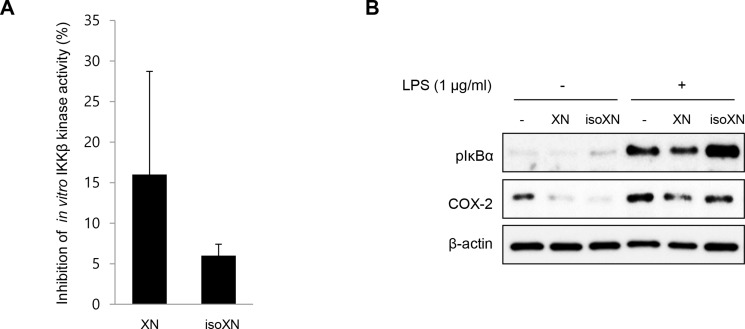
XN inhibits IKKβ kinase activity *in vitro* (**A**) The inhibitory effect of XN (10 μM) and isoXN (10 μM) on IKKβ kinase activity *in vitro* was analyzed by SelectScreen™ Biochemical Kinase Profiling Service. Data represent the mean ± SD of two independent experiments. (**B**) The effects of XN (25 μM) and isoXN (25 μM) on the phosphorylation of IκBα and the expression of COX-2 were determined by Western blot analysis in IEC-6 cells exposed to LPS (1 μg/ml) for 1 h.

Xn and isoXN, a spontaneous cyclization product of XN without electrophilic properties and ability for Michael addition [30], which cause various biological functions such as anti-tumor or anti-inflammatory activity [28, 31]. Therefore, we examined the effect of XN and isoXN on IKKβ/NF-κB signaling *in vitro*. To compare the inhibitory effect of XN and isoXN on IKKβ kinase activity, we assayed the fluorescence-based, coupled-enzyme format IKKβ kinase activity *in vitro*, and we found that the inhibitory effect of XN on the kinase activity of IKKβ was 2 times more than that of isoXN (Figure 1A). In addition, we compared the effects of the aforementioned analogs on the phosphorylation of IκBα and the expression of COX-2 in lipopolysaccharide (LPS)-treated rat intestinal epithelial IEC-6 cells. As shown in Figure 1B, XN exerted more pronounced inhibitory effect on LPS-induced phosphorylation of IκBα and the expression of COX-2 than isoXN. Consistent with these results, it has been reported that XN directly inhibited IKK/NF-κB activation [32].

### XN attenuated DSS-induced acute colitis in mice

**Figure 2:**
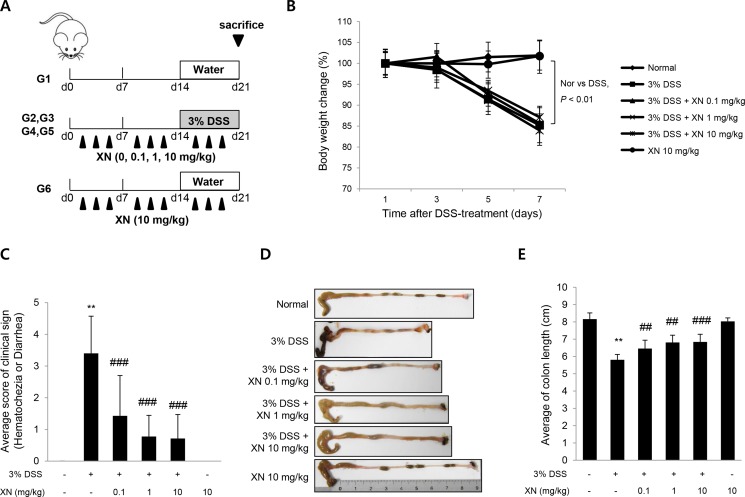
XN decreases the severity of DSS-induced acute colitis in mice (**A**) Diagram shows the experimental protocol of DSS-induced colitis mouse model. (**B**) Body weight changes of all groups (*n* = 10) were measured after DSS induction of colitis. (**C**) Clinical disease activity index were observed after DSS treatment. (**D**) Representative images of the colons and (**E**) colon lengths of the mice were measured. The data represent mean ± SD (*n* = 10); ^**^P < 0.01 *vs.* normal group; ^##^*P* < 0.01 and ^###^*P* < 0.001 *vs.* DSS group.

**Figure 3:**
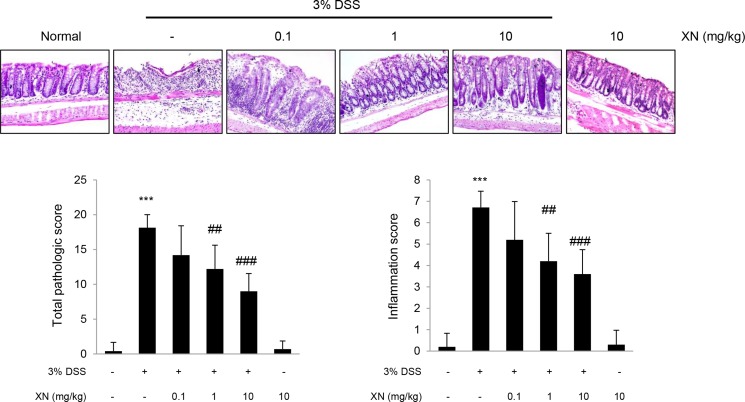
XN prevents DSS-induced intestinal damage in mice Paraffin sections of colons were stained with hematoxylin and eosin (H&E, magnification, × 100). Total pathological score and inflammation score in each group were quantified from H&E-stained sections (bottom). The data represent mean ± SD (*n* = 10); ^***^P < 0.001 *vs.* normal group; ^##^*P* < 0.01 and ^###^*P* < 0.001 *vs.* DSS group.

**Figure 4:**
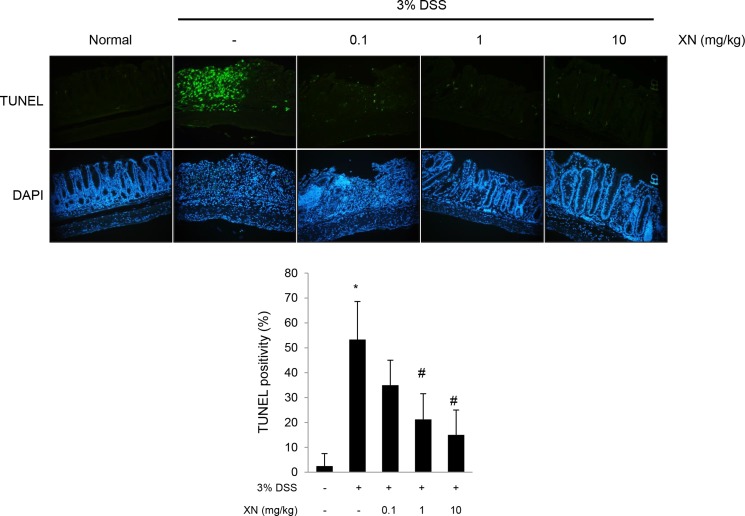
XN inhibits DSS-induced intestinal injury in mice Sections from colonic tissue were stained with terminal deoxynucleotide transferase dUTP nick-end labeling (TUNEL) staining kit (magnification, × 100). The sections were counterstained with DAPI. TUNEL positive cells were counted and scored. The data represent mean ± SD (*n* = 4); ^*^P < 0.05 *vs.* normal group; ^#^*P* < 0.05 *vs.* DSS group.

To explore the anti-inflammatory effect of XN *in vivo*, we used DSS-induced colitis mouse model. XN (0.1, 1 or 10 mg/kg) was orally administered three times a week for two weeks before 3% (w/v) DSS treatment and for a week with DSS exposure (Figure 2A). Administration of DSS by drinking water induced significant disease symptoms such as body weight loss (Figure 2B), clinical signs including hematochezia and diarrhea (Figure 2C) and shortening of colon length (Figure 2D and 2E) when compared to normal group (water group). Pretreatment with XN showed preventive effects on these severe clinical parameters in a dose-dependent manner. Histopathological observation showed that DSS treatment induced the severe inflammation, considerable destruction of mucosal layer and loss of crypts. The severity of DSS-induced colonic damage was determined with total pathologic score and inflammation score, reflecting disruption of the epithelial architecture with a loss of crypts and epithelial integrity, and infiltration of inflammatory cells (Figure 3). However, pretreatment with XN protected the colonic architecture and inhibited inflammatory cell infiltration dose-dependently. To investigate whether intestinal injury induced by DSS is associated with apoptotic cell death, we performed terminal deoxynucleotidyl transferase-mediated dUTP nick-end labeling (TUNEL) staining, a classic indicator of apoptotic cells. As expected, colon tissues in DSS group showed a high number of apoptotic cells whereas TUNEL-positive cells were significantly decreased in XN-pretreated mice in a dose-dependent way (Figure 4). Taken together, these results suggested that XN protects DSS-induced colitis in mice.

### XN inhibited pro-inflammatory responses in DSS-induced colitis in mice

**Figure 5:**
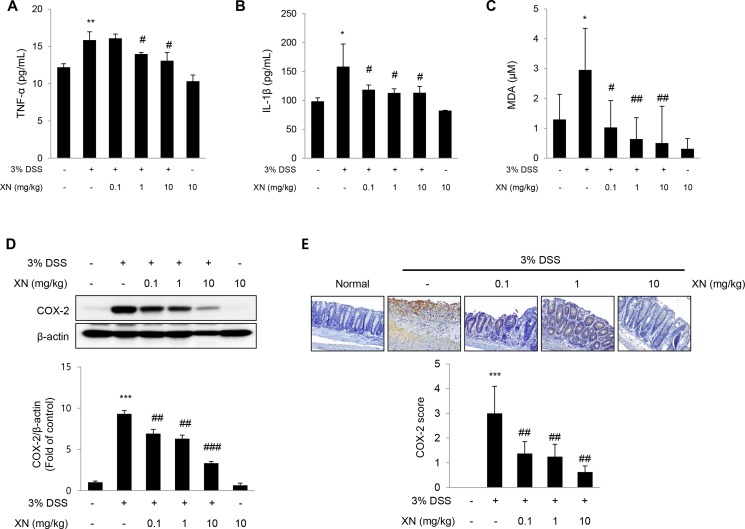
XN inhibits pro-inflammatory responses in DSS-induced colitis *in vivo* The levels of pro-inflammatory cytokines, TNF-α (**A**) and IL-1β (**B**) were measured in serum by ELISA. (**C**) Lipid peroxidation was determined by the measurement of MDA levels in serum. (**D**) The expression of COX-2 was analyzed by Western blot analysis in colon tissues. (**E**) Colon sections were immunostained with anti-COX-2 antibody, and one representative experiment of three was presented (× 100). Counterstaining was done with hematoxylin (blue staining). Staining intensity was scored as 1 for 5% or fewer, 2 for 5% to 20%, 3 for 20% to 50%, 4 for 50% to 80%, and 5 for 80% or more stained cells. The data represent mean ± SD (*n* = 3); ^*^P < 0.05, ^**^P < 0.01 and ^***^P < 0.001 *vs.* normal group; ^#^*P* < 0.05, ^##^*P* < 0.01 and ^###^*P* < 0.001 *vs.* DSS group.

To evaluate the effects of XN on pro-inflammatory responses in DSS-induced colitis, we analyzed the levels of pro-inflammatory cytokines and cyclooxygenase-2 (COX-2). It has been reported that the over-production of pro-inflammatory cytokines can cause oxidative stress, thereby exacerbating DSS-induced colitis [33, 34]. Also, oxidative stress can impair intestinal epithelial cell homeostasis, thereby inducing apoptosis or proliferation. As shown in Figure 5A–5C, the secretory serum levels of tumor necrosis factor (TNF)-α, interleukin (IL)-1β and malondialdehyde (MDA), a lipid peroxidation marker were significantly increased after DSS treatment, while pretreatment with XN decreased the elevated levels of these pro-inflammatory cytokines and oxidative stress in a dose-dependent manner. Even pretreatment with 0.1 mg/kg of XN markedly decreased the levels of TNF-α, IL-1β and MDA compared to the mice given DSS alone. COX-2 is a representative pro-inflammatory mediator in gastrointestinal damages, by which several drugs or strategy had been tried to prevent various gastrointestinal ulcers [35]. To determine whether the preventive effect of XN on DSS-induced colitis is caused by inhibiting the expression of COX-2, we confirmed the expression of COX-2 with Western blot analysis and immunohistochemical staining (Figure 5D and 5E). The expression of COX-2 was markedly increased in DSS-treated mice compared with the untreated mice, whereas pretreatment with XN dramatically decreased the expression of COX-2 induced by DSS challenge. These results demonstrated that XN showed anti-inflammatory effect on DSS-induced colitis *in vivo* through inhibiting the secretion of TNF-α, IL-1β and MDA and the expression of COX-2.

### XN inhibited NF-κB and MAPKs signaling in DSS-induced colitis in mice

**Figure 6:**
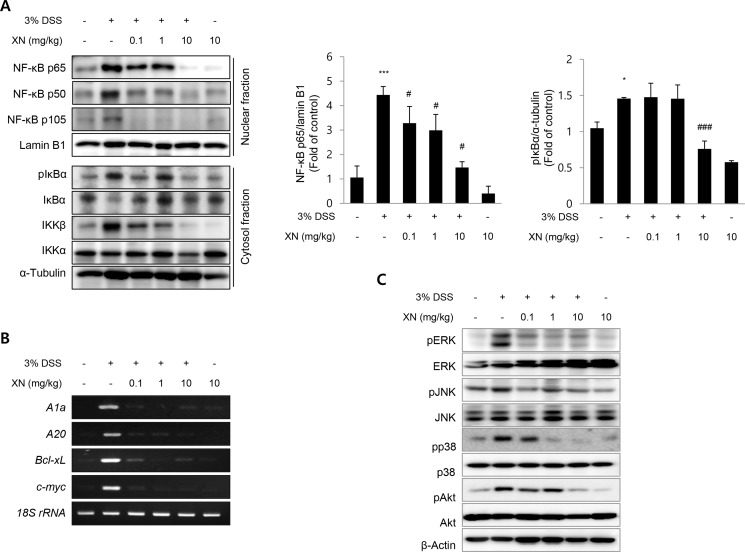
XN inhibits NF-κB and MAPKs signaling in DSS-induced colitis *in vivo* (**A**) The nuclear expressions of p65, p50, p105, and lamin B1 and the cytosolic expressions of pIκBα, IκBα, IKKβ, IKKα and α-tubulin were analyzed by Western blot analysis. (**B**) The mRNA expressions of *A1a*, *A20*, *Bcl-xL*, and *c-myc* in colonic tissues were examined by RT-PCR. 18S rRNA was used as an internal control for the expression of NF-κB target genes. (**C**) The expression of pERK, ERK, pJNK, JNK, pp38, p38, pAkt and Akt in colons were determined by Western blotting. β-actin was used as an internal control. The data represent mean ± SD (*n* = 3); ^*^P < 0.05 and ^***^P < 0.001 *vs.* normal group; ^#^*P* < 0.05 and ^###^*P* < 0.001 *vs.* DSS group.

NF-κB is a key regulator of immune responses and the activation of NF-κB is considered as an important point in the development of colitis [36]. The production of pro-inflammatory cytokines and enzymes such as COX-2 is mediated by NF-κB activation [37]. Based on this rationale, we hypothesized that the anti-inflammatory effect of XN in DSS-induced colitis may correlate with the inhibition of NF-κB signaling. As shown in Figure 6A, the nuclear translocation of p65, p50 and p105 was significantly increased in DSS-exposed mice while XN pretreatment abrogated the translocation of these subunits to the nucleus. Furthermore, the DSS-induced phosphorylation/degradation of IκBα, hallmarks of canonical NF-κB activation was significantly inhibited by pretreatment with XN in the cytosol fraction. To confirm the inhibitory effect of XN on NF-κB signaling, we investigated the mRNA expressions of NF-κB downstream target genes, *A1a*, *A20*, *Bcl-xL*, and *c-myc*. As expected, DSS challenge induced the expression of these genes, which were remarkably reduced in all XN-pretreated groups (Figure 6B). These results suggested that XN significantly blocks the NF-κB signaling pathway in DSS-induced colitis by suppressing IκBα phosphorylation, blocking the nuclear translocation of NF-κB p65, p50 and p105, and inhibiting NF-κB downstream signaling.

Mitogen-activated protein kinases (MAPKs) pathway has been reported to participate in the regulation of NF-κB activation [38]. Recent study demonstrated that DSS induced the activation of ERK, JNK, and p38 in the inflamed colon [39]. To investigate whether MAPKs implicates in the regulatory effects of XN on DSS-induced inflammation, we assessed the activation of ERK, JNK, and p38 MAPKs in DSS-induced colitis. As shown in Figure 6C, the phosphorylation of ERK, JNK and p38 was activated in DSS-induced colon tissues. However, XN pretreatment attenuated the phosphorylation of ERK, JNK and p38 MAPKs in DSS-induced colon tissues in a dose-dependent manner while no changes in the total form. The activation of PI3K/Akt pathway is also important in colitis and can activate the downstream target of NF-κB through the phosphorylation of IKK complex [40, 41]. The phosphorylation of Akt induced by DSS treatment was also dose-dependently decreased in XN-pretreated group (Figure 6C). The results suggested that the suppression of MAPKs and Akt signaling may also contribute to the anti-inflammatory effect of XN.

### XN inhibited NF-κB signaling *in vitro*

**Figure 7:**
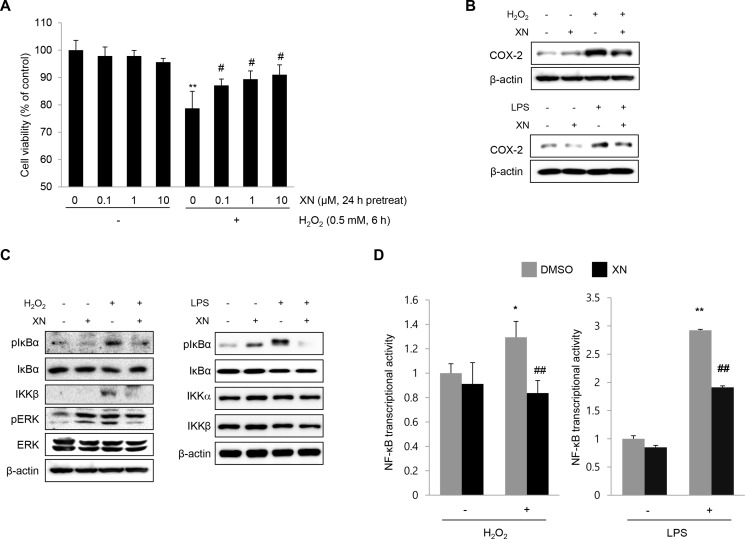
XN attenuates NF-κB signaling in IEC-6 cells (**A**) IEC-6 cells were pretreated with the indicated concentrations of XN for 24 h and treated H_2_O_2_ (0.5 mM) for 6 h. MTT assay was performed for the measurement of cell viability. (**B**) The effect of XN on the expression of COX-2 was analyzed by Western blot analysis in H_2_O_2_- or LPS (1 μg/ml)-treated IEC-6 cells. (**C**) The effect of XN on the expression of pIκBα, IκBα, IKKα, IKKβ, pERK and ERK was analyzed by Western blot analysis in H_2_O_2_- or LPS (1 μg/ml)-treated IEC-6 cells. (**D**) IEC-6 cells were transfected with NF-κB-Luc plasmids. At 24 h after transfection, IEC-6 cells were treated with or without LPS (or H_2_O_2_) for 6 h, in the presence or absence of XN (25 μM) and then performed a reporter assay for the transcriptional activity of NF-κB. Data are representative of three independent experiments. The data represent mean ± SD; ^*^P < 0.05 and ^**^P < 0.01 *vs.* vehicle control; ^##^*P* < 0.01 *vs.* H_2_O_2_- or LPS-treated cells.

To confirm the protective effects of XN against DSS-induced colitis *in vivo*, we investigated the effects of XN on H_2_O_2_- or LPS-treated rat intestinal epithelial IEC-6 cells. First, we determined the cytotoxicity of XN and observed no cytotoxicity up to 10 μM of XN treatment for 24 h in IEC-6 cells (Figure 7A). In addition, pretreatment with XN restored the viability of IEC-6 cells damaged by H_2_O_2_ treatment (Figure 7A). In accordance with the *in vivo* data, XN inhibited the expression of COX-2 and phosphorylation of IκBα induced by H_2_O_2_ or LPS in IEC-6 cells (Figure 7B and 7C). Moreover, induction of NF-κB-mediated luciferase activity in IEC-6 cells treated by H_2_O_2_ or LPS was inhibited by XN pretreatment (Figure 7D). These results indicated that XN can protect against H_2_O_2_- or LPS-induced cell damage and inflammation by inhibiting NF-κB activation such as suppression of IκBα degradation and NF-κB-mediated transcriptional activity.

### Molecular docking analysis predicted the covalent interaction between α, β-unsaturated carbonyl moiety of XN and Cys99 of IKKβ

**Figure 8:**
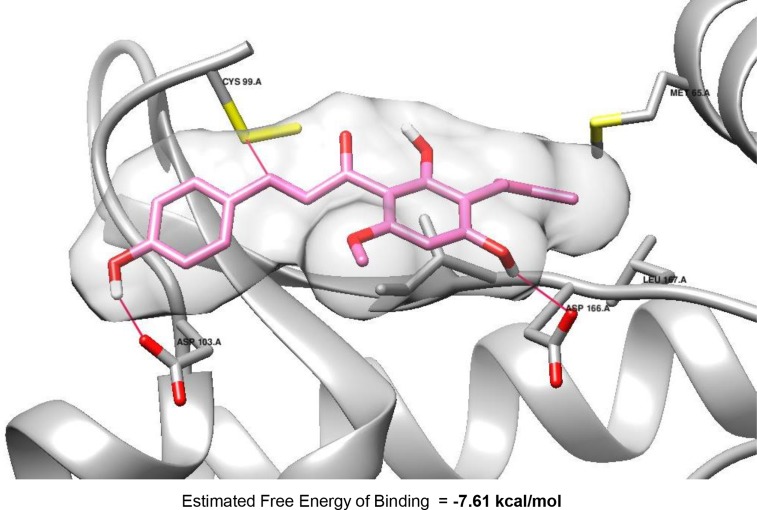
Molecular docking model of XN (pink) with IKKβ (PDB Code: 4KIK), which was visualized using Chimera 1.10 (UCSF Chimera)

To investigate the binding mode of XN, docking analysis of the compound within IKKβ active site was performed using Autodock 4.2 (Molecular Graphic Laboratory) [3, 42]. The crystal structure of IKKβ complexed with K252a, non-selective kinase inhibitor was used for this study. As illustrated in Figure 8, XN fitted well into the active site, which concurred with its potent IKKβ inhibitory activity. The estimated free binding energy of XN to IKKβ is –7.61 kcal/mol. It indicated that the complex of IKKβ and fitted XN might be energetically favored. It is plausible that the both *para* phenol moieties in XN interact with Asp103 and Asp166, respectively, through hydrogen bonds whereas the dimethyl allyl moiety of the compound is oriented toward a lipophilic region surrounded by Met65. Especially, in the complex, the electrophilic β site of α, β-unsaturated carbonyl moiety in the conformation might be positioned near Cys99 that have an ability to do nucleophilic attack, suggesting the possibility of forming a covalent bond between Cys99 and the electrophilic β carbon of enone moiety, leading to significant inhibition of the kinase activity. Additional study of the interaction is being performed by our group and related reports will be addressed near future.

## DISCUSSION

UC is chronic inflammatory disorder affecting the mucosal surface in the colon and small intestine, which occurs predominantly in western countries. However, the prevalence of IBD in developing countries is also increasing steadily [43]. The impaired immunological balance has been reported in gastrointestinal tract of IBD patients, which triggers pro-inflammatory cytokines release and immune cells recruitment, resulting in colonic damage [1]. The IKK/NF-κB signaling is critical for maintenance of immunological balance in the gastrointestinal tract [2]. To find an effective natural compound to block NF-κB signaling pathway, we performed IKKβ kinase activity assay with in-house natural product library and we have excavated XN as a prominent candidate to decrease IKKβ activity in the tested library. There are many reports of the protective effects of XN on several diseases such as inflammation and cancers in various organs [18–25]. Even though it has been reported that the anti-inflammatory effect of XN through NF-κB signaling, the molecular mechanism and function of XN has not yet been examined in DSS-induced colitis mouse model. In the current study, we investigated the protective effect of XN against DSS-induced colitis and found that XN ameliorated DSS-induced colitis through inhibition of NF-κB signaling by interacting with an IKKβ.

To explore the preventive effect of XN *in vivo*, we evaluated the anti-inflammatory effect of XN using the acute colitis model induced by DSS in C57BL/6 mice.After pretreatment of XN in DSS-treated mice, XN relieved the colitis symptoms such as diarrhea, hematochezia, rectal bleeding and shortening of colon length (Figure 2). In addition, XN prevented destruction of the epithelial architecture with infiltration of immune cells and a loss of crypts by DSS challenge (Figure 3). During the progression of UC, the inflammatory signaling impairs the intestinal epithelial function and leads to the recruitment of inflammatory cells and inflammatory cytokines to the site of injury [44]. DSS-induced high-production of pro-inflammatory cytokines, TNF-α and IL-1β was significantly inhibited by the pretreatment with XN (Figure 5A and 5B). In addition, the preadministration of XN caused a marked decrease of MDA levels induced by DSS exposure (Figure 5C), in agreement with anti-oxidative effect of XN in the previous study [28]. Furthermore, the pretreatment with XN reduced the expression of COX-2 in this study (Figure 5D and 5E). As a representative pro-inflammatory enzymes in colitis, COX-2 has been known to involve in production of oxidative stress and induction of pro-inflammatory cytokines [35]. Therefore, these results suggest that the protective effect of XN could be conferred by inhibiting the generation of pro-inflammatory cytokines, oxidative stress and the overexpression of COX-2 in DSS-induced colitis.

Inflammation mediated by high concentration of pro-inflammatory cytokines and enzymes has been regulated by the activation of transcriptional factors such as NF-κB in IBD patients [13, 14, 36]. Many studies have reported that NF-κB activation induces the expression of target genes including inflammation and carcinogenesis, promoting cell survival, proliferation and angiogenesis [8–10]. Our results indicate that XN treatment inhibited the phosphorylation of IκBα, the nuclear translocation of p65, p50 and p105 subunits and the DNA-binding transcriptional activity of NF-κB in DSS-treated mice and H_2_O_2_- or LPS-treated IEC-6 cells (Figures 6 and 7). Moreover, we confirmed that XN abrogated the expression of NF-κB target genes such as *A1a*, *A20*, *Bcl-xL*, and *c-myc*, indicating the inhibitory effect of XN on the activation of canonical NF-κB pathway. Consistent with our results, XN has been reported to inhibit the activation of NF-κB in prostate epithelial cells [25] and endothelial cells [27]. The activation of NF-κB requires IKK complex [10], so we investigated the expression of IKKα and IKKβ and found that XN significantly reduced the expression of IKKβ *in vivo* and *in vitro*, resulting in the decrease of phosphorylation/degradation of IκBα. The two IKKs have similar structure but perform different function. IKKβ has been known to regulate the canonical NF-κB activation whereas IKKα is considered to be dispensable for the canonical NF-κB pathway [12]. Therefore, we examined the effect of XN on the kinase activity of IKKβ and the downstream signaling *in vitro*. As expected, XN inhibited the kinase activity of IKKβ, the phosphorylation of IκBα and the expression of COX-2 in LPS-treated IEC-6 cells.

XN is a part of our diet mainly in the form of beer or beer mix drinks. XN is easily converted to the flavanone isoXN during the brewing process because isoXN is a spontaneous cyclization product of XN. Comparing anti-inflammatory effects of XN with isoXN in terms of suppression of IKKβ activity and the phosphorylation of IκBα (Figure 1), XN was found to be more effective in suppressing IKKβ/NF-κB signaling than isoXN. Investigation of the chemical structure of XN identifies that the compound contains one α, β-unsaturated carbonyl moiety, a thiol-reactive electrophilic center that can covalently interact with the thiols of cysteine residue. In line with this notion, our docking analysis reveals that XN can bind to the active site of IKKβ and it is energetically favored (Figure 8). The electrophilic β site of α, β-unsaturated ketone in the conformation in the XN-IKKβ complex might be placed near Cys99 that subsequently induce nucleophilic attack, suggesting the possibility of forming a covalent bond between Cys99 and the electrophilic β carbon of enone moiety, leading to significant inhibition of the kinase activity. These results suggest that XN might inhibit IKKβ/NF-κB signaling through direct interaction with cysteine thiol of IKKβ, thereby suppressing the downstream of NF-κB signaling. In support with these results, XN has been reported to directly inhibit the activation of IKK/NF-κB pathway [32]. In this study, XN reduced the expression of IKKβ at protein level *in vivo* and *in vitro* (Figures 6 and 7), suggesting that XN may decrease the transcriptional expression of IKKβ or the proteasomal degradation of IKKβ. Recent paper has reported that Cullin 3-based ubiquitin ligase, KEAP1 is involved in the dysregulation/ubiquitination of IKKβ in tumorigenesis [45]. Interestingly, KEAP1 also has cysteine thiols, implying that XN may be a binding partner of KEAP1 as well as IKKβ. We are now exploring this possibility of the interaction with new *in vivo* studies in mice treated by AOM/DSS.

In conclusion, our study provides that XN has protective effects against DSS-induced colitis in mice and H_2_O_2_- or LPS-treated IEC-6 cells by inhibiting IKKβ/NF-κB signaling pathway, indicating the possible interaction between electrophilic carbon center of α, β-unsaturated carbonyl moiety in XN and Cys99 in IKKβ. Taken together, XN could be the potential therapeutic agent for the prevention or treatment of colitis.

## MATERIALS AND METHODS

### Synthesis of xanthohumol

#### 1-(2-hydroxy-4,6-bis(methoxymethoxy)phenyl)ethanone (2)

To a stirred solution of **1** (4.0 g, 23.8 mmol) in dichloromethane (80 mL) was added Et_3_N (13.2 mL, 95.2 mmol, 4.0 eq.) and MOMCl (5.4 mL, 71.4 mmol, 3.0 eq.) at 0°C. The reaction mixture was warmed to room temperature and stirred for 8 h. Water (20 mL) was added to the reaction mixture at 0°C and organic layer was separated. Aqueous layer was extracted with dichloromethane (50 mL, two times) and combined organic layer was washed with water (10 mL, two times). Organic layer was dried over MgSO_4_ and solvent was removed under reduced pressure. The crude material was purified by silica gel column chromatography (EtOAc/hexanes = 1/5) to afford **2** as a yellowish oil (3.35 g, 13.1 mmol, 55%). ^1^H-NMR (CDCl_3_, 300 MHz) *δ* 13.71 (s, 1H), 6.23 (d, 1H, *J* = 2.2 Hz), 6.21 (d, 1H, *J* = 2.2 Hz), 5.23 (s, 2H), 5.14 (s, 2H), 3.49 (s, 3H), 3.44 (s, 3H), 2.62 (s, 3H).

#### 1-(2,4-bis(methoxymethoxy)-6-((3-methylbut-2-en-1-yl)oxy)phenyl)ethanone (3)

To a solution of **2** (3.2 g, 12.5 mmol) in acetone (80 mL) was added K_2_CO_3_ (6.91 g, 50.0 mmol, 4.0 eq.), prenyl bromide (2.2 mL, 18.8 mmol, 1.5 eq.) then the reaction mixture was heated to reflux for 24 h. Volatiles were removed under reduced pressure and water (20 mL) and EtOAc (150 mL) was added. Organic layer was separated and washed with water (10 mL, two times). Organic layer was dried over MgSO_4_ and solvent was removed under reduced pressure. The crude material was purified by silica gel column chromatography (EtOAc/hexanes = 1/5) to afford **3** as a colorless oil (3.28 g, 10.1 mmol, 81%). ^1^H-NMR (CDCl_3_, 300 MHz) *δ* 6.41 (d, 1H, *J* = 1.8 Hz), 6.28 (d, 1H, *J* = 1.8 Hz), 5.36 (t, 1H, *J* = 6.6 Hz), 5.12 (s, 2H), 5.08 (s, 2H), 4.46 (d, 2H, *J* = 6.6 Hz), 3.44 (s, 3H), 3.42 (s, 3H), 2.44 (s, 3H), 1.73 (s, 3H), 1.67 (s, 3H).

#### 1-(6-hydroxy-2,4-bis(methoxymethoxy)-3-(3-methylbut-2-en-1-yl)phenyl)ethanone (4)

To a round-bottomed flask **3** (2.54 g, 7.83 mmol) and *N*,*N*-diethylaniline (DEA, 60 mL) was added and heated to reflux for 24 h. Remaining DEA was removed by short path distillation apparatus under high vacuum conditions. The crude material was purified by silica gel column chromatography (EtOAc/hexanes = 1/5) to afford **4** as a colorless oil (1.14 g, 3.52 mmol, 45%). ^1^H-NMR (CDCl_3_, 400 MHz) *δ* 12.90 (s, 1H), 6.44 (s, 1H), 5.18 (s, 2H), 5.12 (m, 1H), 4.93 (s, 2H), 3.49 (s, 3H), 3.43 (s, 3H), 3.28 (d, 2H, *J* = 6.5 Hz), 2.67 (s, 3H), 1.74 (s, 3H), 1.66 (s, 3H).

#### 1-(6-methoxy-2,4-bis(methoxymethoxy)-3-(3-methylbut-2-en-1-yl)phenyl)ethanone (5)

To a solution of **4** (1.2 g, 3.70 mmol) in acetone (70 mL) was added K_2_CO_3_ (1.53 g, 11.1 mmol, 3.0 eq.), iodomethane (0.46 mL, 7.40 mmol, 2.0 eq.) then the reaction mixture was heated to reflux for 24 h. Volatiles were removed under reduced pressure and water (10 mL) and EtOAc (150 mL) was added. Organic layer was separated and washed with water (10 mL, two times). Organic layer was dried over MgSO_4_ and solvent was removed under reduced pressure. The crude material was purified by silica gel column chromatography (EtOAc/hexanes = 1/5) to afford **5** as a colorless oil (1.06 g, 3.15 mmol, 85%). ^1^H-NMR (CDCl_3_, 400 MHz) *δ* 6.52 (s, 1H), 5.18 (s, 2H), 5.11 (t, 1H, *J* = 6.7 Hz), 4.88 (s, 2H), 3.77 (s, 3H), 3.46 (s, 3H), 3.45 (s, 3H), 3.27 (d, 1H, *J* = 6.7 Hz), 2.47 (s, 3H), 1.72 (s, 3H), 1.63 (s, 3H).

#### (E)-1-(6-methoxy-2,4-bis(methoxymethoxy)-3-(3-methylbut-2-en-1-yl)phenyl)-3-(4-(methoxymethoxy)phenyl)prop-2-en-1-one (7)

To a solution of **5** (1.2 g, 4.33 mmol) in EtOH (50 mL) was added KOH (486 mg, 8.66 mmol, 2.0 eq.), 4-(methoxymethoxy)benzaldehyde (1.08 g, 6.50 mmol, 1.5 eq.) then the reaction mixture was heated to 50°C for 10 h. Volatiles were removed under reduced pressure and water (10 mL) and EtOAc (150 mL) was added. Organic layer was separated and washed with water (10 mL, two times). Organic layer was dried over MgSO_4_ and solvent was removed under reduced pressure. The crude material was purified by silica gel column chromatography (EtOAc/hexanes = 1/5) to afford **7** as a yellow oil (1.54 g, 3.16 mmol, 73%). ^1^H-NMR (CDCl_3_, 300 MHz) *δ* 7.41 (d, 1H, *J* = 8.8 Hz), 7.30 (d, 1H, *J* = 15.9 Hz), 6.95 (d, 1H, *J* = 8.8 Hz), 6.83 (d, 1H, *J* = 15.9 Hz), 6.51 (s, 1H), 5.16 (s, 2H), 5.13 (s, 2H), 5.11–5.03 (m, 1H), 4.83 (s, 2H), 3.68 (s, 3H), 3.43 (s, 3H), 3.40 (s, 3H), 3.35 (s, 3H), 3.27 (d, 1H, *J* = 6.8 Hz), 1.69 (s, 3H), 1.61 (s, 3H).

#### (E)-1-(2,4-dihydroxy-6-methoxy-3-(3-methylbut-2-en-1-yl)phenyl)-3-(4-hydroxyphenyl)prop-2-en-1-one (8)

To a solution of **7** (650 mg, 1.34 mmol) in MeOH (50 mL) was added *c*-HCl (5 drops) then the reaction mixture was heated to reflux for 5 h with TLC monitoring. Volatiles were not removed because considerable amount of product were cyclized in acidic condition during evaporation of MeOH. Water (10 mL) and EtOAc (150 mL) was directly added to the reaction mixture. Organic layer was separated and washed with water (10 mL, three times for removal of remaining HCl). Organic layer was dried over MgSO_4_ and solvent was removed under reduced pressure. The crude material was purified by silica gel column chromatography (EtOAc/hexanes = 1/2 to 1/1) to afford **8** as a yellow solid (294 mg, 0.83 mmol, 62%). ^1^H-NMR (CDCl_3_, 300 MHz) *δ* 14.63 (s, 1H), 7.78 (d, 1H, *J* = 15.5 Hz), 7.72 (d, 1H, *J* = 15.5 Hz), 7.49 (d, 2H, *J* = 8.6 Hz), 6.84 (d, 2H, *J* = 8.6 Hz), 6.16 (bs, 1H), 5.92 (s, 1H), 5.28 (t, 1H, *J* = 7.2 Hz), 5.09 (s, 1H), 3.88 (s, 3H), 3.38 (d, 2H, *J* = 7.1 Hz), 1.81 (s, 3H), 1.76 (s, 3H).

#### 7-hydroxy-2-(4-hydroxyphenyl)-5-methoxy-8-(3-methylbut-2-en-1-yl)chroman-4-one (9)

To a round-bottomed flask **8** (45 mg, 0.127 mmol) in MeOH was added aqueous 1.0 M NaOH solution and stirred for 10 h. Diluted HCl (1.0 M solution) was added to the reaction mixture for acidification. The reaction mixture was extracted with EtOAc for three times (50 mL). Combined organic layer was separated and washed with water (10 mL, two times). Organic layer was dried over MgSO_4_ and solvent was removed under reduced pressure. The crude reaction mixture was purified by silica gel column chromatography (EtOAc/hexanes = 1/1) to afford **9** as a white solid (29.3 mg, 0.0826 mmol, 65%). ^1^H-NMR (CDCl_3_, 300 MHz) *δ* 7.24 (d, 2H, *J* = 8.6 Hz), 6.80 (d, 2H, *J* = 8.6 Hz), 6.20 (s, 1H), 6.01 (s, 1H), 5.27 (dd, 1H, *J* = 12.5, 3.0 Hz), 5.17–5.14 (m, 2H), 3.78 (s, 3H), 3.29 (d, 2H, *J* = 7.1 Hz), 2.94 and 2.89 (d, 1H, *J* = 12.6 Hz), 2.76 and 2.71 (d, 1H, *J* = 3.1 Hz), 1.683 (s, 3H), 1.682 (s, 3H).

### Reagents and antibodies

XN was dissolved in dimethylsulfoxide (DMSO) as stock solution and stored at –20°C. DMSO was purchased from Sigma Chemical Co. (St. Louis, MO). Dextran sulfate sodium (DSS, molecular weight 36–50 kDa) was purchased from MP Biomedicals Inc. (Irvine, CA, USA). NE-PER™ Nuclear and Cytoplasmic Extraction Reagents was purchased from Thermo Fisher Scientific, Inc. (Waltham, MA). Antibodies for p65, p50, p105, p-ERK, ERK, p-JNK, JNK, β-actin and α-tubulin were purchased from Santa Cruz Biotechnology (Santa Cruz, CA, USA). Antibodies for IKKα, IKKβ, p-IkBα, IkBα, p-p38, p38, p-Akt and Akt were purchased from Cell Signaling Technology (Danvers, MA). Antibodies for COX-2 and Lamin B1 were purchased from Thermo Fisher Scientific, Inc. (MA, USA).

### Mice and DSS-induced acute colitis model

Six-week-old male C57BL/6 mice in the experiment were purchased from Orient bio (Seoul, Korea). All animals were housed in an experimental room at 12 hours of light/dark cycle at 24°C with ad libitum access to water and a rodent chow diet under specific pathogen-free conditions. The animals were handled at accredited animal facilities in accordance with the Institutional Animal Care and Use Committee (IACUC) of the CHA University Animal Center (reference number: IACUC150123). After acclimatization, healthy seven-week-old mice (18 ∼ 22 g) were used for the experiments.

The DSS-induced colitis animal model exhibits many phenotype that are relevant to human UC, including inflammation and ulceration of the colonic mucosa [46]. Acute colitis was induced by administration of DSS in drinking water for a week. Seven-week-old mice (18 ∼ 22 g) were randomly divided into 6 groups (*n* = 10); a vehicle control, 3% (w/v) DSS-treated, DSS+XN (0.1, 1 and 10 mg/kg mouse body weight)-treated and XN (10 mg/kg) -treated groups. XN was pretreated for 2 weeks before DSS treatment and treated for a week with DSS exposure. XN was dissolved in 50% DMSO and the remaining 50% was added with water and administered orally 3 times per week for three weeks (Figure 2A). Clinical phenotypes such as hematochezia and diarrhea were investigated and charted daily. After 7 days of DSS ingestion, all mice were sacrificed and colons were removed, rinsed with PBS and the lengths of colons were measured.

### Histopathological and immunohistochemical analysis of colonic lesions

For histopathological assessment, fixed colon portions were embedded in paraffin blocks, followed by cutting 4 μm sections and mounting them on glass slides for hematoxylin-eosin (H&E) staining. The mean pathological index was calculated using criteria described in [47]. Briefly, the pathological changes including intensity of ulceration and inflammatory cells infiltration were observed. For immunohistochemical assessment, 4 μm paraffin-embedded colon sections were mounted on coated glass slides for detection of proteins under investigation. Following antigen retrieval and blocking endogenous peroxidases and nonspecific protein binding, slide sections were incubated first with the primary antibodies, followed by HRP-conjugated secondary antibodies. Then, the chromogen was added for color development. The primary antibodies for COX-2 (diluted 1:100) were purchased from Cayman chemical (Ann Arbor, MI, USA). All slides were developed with 3,3’ diaminobenzidine followed by hematoxylin counterstaining. Scoring was done by the corresponding author, who was blinded to the primary antibodies and the treatment groups. For COX-2, the percentage of positively stained cells was estimated. Cases with 5% or fewer positively stained cells were scored as 1, 2 for 5%–20%, 3 for 20%–50%, 4 for 50%–80%, and 80% or more stained cells were denoted as 5.

### RNA isolation and reverse transcription polymerase chain reaction (RT-PCR)

Total RNA was extracted from the colons using RiboEX (GeneAll, Seoul, Korea) and complementary DNA was prepared using M-MLV reverse transcriptase using SuperScript™ II Reverse Transcriptase kit (Invitrogen, Waltham, MA) according to the manufacturer’s instructions. RT-PCR was performed as previously reported [48] and assessed for 30 cycles at 94°C for 20 seconds, 58°C for 30 seconds, and 72°C for 45 seconds. Oligonucleotide primers were as follows: Ala: sense 5′- CCT GGC TGA GCA CTA CCT TC-3′, antisense 5′-TTC TGC CGT ATC CAT TCT CC-3′; A20: sense 5′-AGC TAG GCC CTG AAG GAC TC-3′, antisense 5′-CTT GTC CCT GCT CTG TCT CC-3′; Bcl-xL: sense 5′-TGG TGG TCG ACT TTC TCT CC-3′, antisense 5′-TGC AAT CCG ACT CAC CAA TA-3′; c-myc: sense 5′- TGC GAC GAG GAA GAG AAT TT -3′, antisense 5′- AAC CGC TCC ACA TAC AGT CC -3′; 18s rRNA: sense 5′-CCC AAC TTC TTA GAG GGA CAA GT-3′, antisense 5′-TAG TCA AGT TCG ACC GTC TTC TC-3′

### Western blot analysis

The colon tissues were homogenized with ice-cold cell lysis buffer containing protease inhibitor (Roche Applied Science, Mannheim, Germany) and centrifuged to remove the pellet and debris. Western blot analysis was performed as previously described [49]. Proteins were separated by SDS-PAGE and transferred to polyvinylidene fluoride membranes, which were incubated with the primary antibodies, washed, incubated with peroxidase-conjugated secondary antibodies, rewashed, and then visualized using an enhanced chemiluminescence system (Thermo Fisher Scientific, MA).

### Cytokines and MDA measurement by enzyme-linked immunoassay (ELISA)

The serum levels of TNF-α, IL-1β and MDA were measured with an enzyme-linked immunosorbent assay kit (mouse Quantikine ELISA kit, R&D Systems, Minneapolis, USA) following the manufacturer’s instruction.

### TdT-mediated biotinylated UTP nick end labeling (TUNEL) assay

To detect damaged cells in colon sections, we performed TUNEL staining according to the manufacturer’s instructions (Roche, Indianapolis, IN). Apoptosis was quantified by assessing TUNEL-positive nuclei staining cells from five to ten selected crypts for each mouse, and the apoptotic index was defined as the percentage of TUNEL-positive cell-containing crypts.

### Cell culture and cytotoxicity assay

Rat intestinal epithelial IEC-6 cells were obtained from the American Type Culture Collection (ATCC, Rockville, MD) and maintained according to the ATCC’s instructions. IEC-6 cells were maintained at 37°C in a humidified atmosphere containing 5% CO_2_ and cultured in Dulbecco’s modified Eagle’s medium (HyClone, GE Healthcare, UT, USA) containing 10% (*v/v*) fetal bovine serum (ATCC), 100 U/ml penicillin and 100 μg/ml streptomycin. IEC-6 cells (1.0 × 10^5^) were plated in 96-well plates and incubated for 24 h after which media was changed with fresh one containing XN. Cell cytotoxicity was measured by 3-(4, 5-dimethylthiazol-2-yl)-2, 5-diphenyltetrazolium bromide (MTT) assay.

### Luciferase assay

IEC-6 cells in 24-well plates were transiently transfected with NF-κB promoter luciferase reporter using Lipofectamine^®^ 2000 Transfection Reagent (Invitrogen, Waltham, MA). Twenty-four hours after transfection, IEC-6 cells were treated with or without LPS (or H_2_O_2_) for 6 h, in the presence or absence of XN. Cells were collected and assayed for the luciferase activity using the luciferase assay system (Promega, Madison, WI) according to the manufacturer’s instructions. Each experiment was repeated in triplicate.

### IKKβ kinase activity analysis

IKKβ kinase activity was accomplished using SelectScreen™ Biochemical Kinase Profiling Service provided Thermo Fisher Scientific (Waltham, MA). The Z´-LYTE biochemical assay employed a fluorescence-based, coupled-enzyme format and is based on the differential sensitivity of phosphorylated and non-phosphorylated peptides to proteolytic cleavage. The peptide substrate was labeled with two fluorophores - one at each end - that make up a FRET pair. In the primary reaction, the kinase transferred the gamma-phosphate of ATP to a single tyrosine, serine or threonine residue in a synthetic FRET-peptide. In the secondary reaction, a site-specific protease recognized and cleaved non-phosphorylated FRET-peptides. Phosphorylation of FRET-peptides suppressed cleavage by the Development Reagent. Cleavage disrupted FRET between the donor and acceptor fluorophores on the FRET-peptide, whereas uncleaved, phosphorylated FRET-peptides maintain FRET. A ratiometric method, which calculates the ratio (the Emission Ratio) of donor emission to acceptor emission after excitation of the donor fluorophore at 400 nm, was used to quantitate reaction progress, as shown in the equation below.

Emission Ration = Donor Emission (445 nm)/Acceptor Emission (520 nm).

### Docking analysis

The docking parameters consisted of setting the population size to 150, the number of generations to 27,000, and the number of evaluations to 25,000,000, while the number of docking runs was set to 50 with a cutoff of 1 Å for the root-mean-square tolerance for the grouping of each docking run. Docking of XN with Human IκB kinase β (PDB code: 4KIK) was accomplished using the AutoDock 4.2 program downloaded from the Molecular Graphics Laboratory of the Scripps Research Institute. The docking result was demonstrated using Chimera 1.10 (UCSF Chimera).

### Statistical analysis

Results are expressed as the mean ± standard deviation (SD). The statistical significance was analyzed by one-way analysis of variance (ANOVA). Statistical significance was accepted at *P* < 0.05.

## Abbreviations

XN: xanthohumol
isoXN: isoxanthohumol
UC: ulcerative colitis
IBD: inflammatory bowel disease
DSS: dextran sulfate sodium
NF-κB: Nuclear factor-κB
IKK: IκB kinases
TUNEL: terminal deoxynucleotidyl transferase-mediated dUTP nick-end labeling
COX-2: cyclooxygenase-2
TNF-α: tumor necrosis factor-α
IL-1β: interleukin-1β
MDA: malondialdehyde
MAPKs: Mitogen-activated protein kinases
